# Post-resuscitation pre-hospital emergency anaesthesia for exchange of a supraglottic airway device with an endotracheal tube - a multicentre observational study

**DOI:** 10.1186/s12873-026-01668-8

**Published:** 2026-07-13

**Authors:** Jonas Bökamp, Eugen Latka, Michael Bernhard, Martin Deicke, Daniel Fischer, Julia Grannemann, Jochen Hinkelbein, Yacin Keller, Annika Hoyer, André Kobiella, Lydia Johnson Kolaparambil Varghese, Bernd Strickmann, Mathini Vaseekaran, Vera Von Dossow, Gerrit Jansen

**Affiliations:** 1 Medical School OWL, Bielefeld University, Universitätsstraße 25, 33615 Bielefeld, Germany; 2 Department of Medicine and Emergency Services, Westphalia-Lippe Study Institute, Rohrteichstraße 71, 33602 Bielefeld, Germany; 3 Emergency Department, University Hospital Düsseldorf, Moorenstraße 5, 40225 Düsseldorf, Germany; 4 Medical Direction of Emergency Services, Osnabrück Rural District, Am Schölerberg 1, 49082 Osnabrück, Germany; 5 Medical Direction of Emergency Services, Lippe District, Felix-Fechenbach Straße 5, 32756 Detmold, Germany; 6 Medical Direction of Emergency Services, Gütersloh District, Herzebrocker Straße 140, 33334 Gütersloh, Germany; 7 Medical Direction of Emergency Services, Integrated Regional Control Centre, City of Dresden Fire Department, Dr.-Külz-Ring 19, 01067 Dresden, Germany; 8 Biostatistics and Medical Biometry, Medical School OWL, Bielefeld University, Universitätsstraße 25, 33615 Bielefeld, Germany; 9 University Department of Anaesthesiology, Intensive Care Medicine, Emergency Medicine, and Pain Management, Johannes Wesling Hospital Minden, Ruhr-University Bochum, Hans-Nolte Straße 1, 32427 Minden, Germany; 10 Institute of Anaesthesiology and Pain Therapy, Heart and Diabetes Centre NRW, Ruhr-University Bochum, Georgstraße 11, 32545 Bad Oeynhausen, Germany; 11 Institute of Anesthesiology and Pain therapy, Heart and Diabetes Center NRW, University Bielefeld, Medical Faculty OWL, Georgstraße 11, 32545 Bad Oeynhausen, Germany

**Keywords:** Adverse effects, Intubation, Intratracheal, Laryngeal masks, Out-of-hospital cardiac arrest, Post cardiac arrest syndrome

## Abstract

**Background:**

Post-resuscitation pre-hospital emergency anaesthesia (PHEA) for changing a supraglottic airway device (SGA) to an endotracheal tube (ETT) after out-of-hospital cardiac arrest may improve airway management but carries potential risks, particularly related to haemodynamic instability. This study investigates its association with complications during post-resuscitation care.

**Methods:**

All Emergency Medical Service missions in the districts Gütersloh, Dresden and Lippe from 2019 to 2021 were analysed for out-of-hospital cardiac arrest and supplemented with data from the German Resuscitation Register. Unconscious adult, pre-hospitally resuscitated patients who had an SGA changed to an ETT following pre-hospital return of spontaneous circulation and who had spontaneous circulation on admission to hospital were included. We compared patients with and without PHEA. The primary endpoint was the occurrence of haemodynamic complications during post-resuscitation therapy, defined as re-arrest, hypotension or antihypotensive therapy. For statistical analysis, propensity score matching was performed adjusting for age, sex, pre-existing disease, shockable initial cardiac rhythm, bystander CPR and witnessed arrest.

**Results:**

Of 2,305 cardiac arrest patients in 391,305 Emergency Medical Service missions, 706 (30.6%) had return of spontaneous circulation and 202 met our inclusion criteria. 126 of those patients (62.4%) received PHEA to facilitate changing an SGA to an ETT. The propensity score analysis showed no evidence for differences in the odds of occurrence of at least one haemodynamic complication (OR=1.50 [95% CI=0.72-3.11; p=0.28]), airway complication (OR=1.00 [95% CI=0.25-4.00; p=1.00]), re-arrest (OR=0.39 [95% CI=0.14-1.08; p=0.07]), hypotension (OR=1.39 [95% CI=0.68-2.83; p=0.37]) or antihypotensive therapy (OR=1.58 [95% CI=0.77-3.26; p=0.21]) between patients having their SGA changed to an ETT following PHEA compared to patients who had their SGA changed to an ETT without PHEA.

**Conclusions:**

The study provided no statistical evidence for increased odds of complications associated with the delivery or omission of pre-hospital emergency anaesthesia during exchange from a supraglottic airway device to an endotracheal tube in resuscitated out-of-hospital cardiac arrest patients.

**Clinical trial number:**

Not applicable.

**Supplementary Information:**

The online version contains supplementary material available at 10.1186/s12873-026-01668-8.

## Background

Due to its high incidence and mortality out-of-hospital cardiac arrest (OHCA) continues to be a highly important topic in pre-hospital care [1].

In addition to the provision of optimal cardiac life support, the importance of high-quality and targeted post-resuscitation management for the prognosis of affected patients has become apparent in recent years [2, 3].

The implementation of more advanced airway management e.g. by using a supraglottic airway device (SGA) continues to be a topic for research and discussion [4]. Following the intra-arrest use of an SGA current guidelines recommend changing it to an endotracheal tube (ETT) as part of post-resuscitation therapy. Pre-hospital emergency anaesthesia (PHEA) is often used to facilitate this process [3].

PHEA offers advantages such as improved intubation conditions and synchronisation to the ventilator with improved oxygenation and ventilation as well as analgesia and suppression of conscious awareness of the affected patients [5–8]. However, in the immediate phase following pre-hospital return of spontaneous circulation (ROSC) adverse effects of anaesthetics, such as hypotension and re-arrest, could negatively affect the rates of survival and favourable neurologic outcome [9–11].

Emergency anaesthesia and intubation in critically ill patients, both in pre-hospital and in-hospital emergency medicine or in the intensive care unit, has been shown to be a high risk procedure associated with relevant complications and a mortality risk of around 1% in recent years [12]. Although efforts have been made to reduce these complications through various studies and recommendations of the professional societies, the haemodynamically highly vulnerable group of pre-hospital post-resuscitation patients needing PHEA has so far been insufficiently investigated [3, 13–18].

While previous analyses have evaluated PHEA after ROSC in general, the specific clinical situation of replacing an SGA with an ETT represents a distinct and potentially haemodynamically vulnerable setting. Data addressing the safety of PHEA in this specific context are lacking [5, 6].

Against this background, this multicentre study examines the performance and complications of post-resuscitation PHEA for changing an SGA to an ETT using a propensity score analysis.

## Methods

### Ethics approval and consent to participate

Ethical approval for this study was obtained from the Ethics Committee of the Medical Association of Westphalia-Lippe on October 3, 2022 (Chair: Univ.-Prof. Dr. med. Wolfgang E. Berdel; reference 2022-617-f-S). The requirement for informed consent was waived by the ethics committee due to the retrospective design. The study was conducted in accordance with the principles of the Declaration of Helsinki, as revised in 2024.

### Study design and settings

This study was conducted within the German emergency medical services (EMS) system, a physician-based model in which pre-hospital care for critically ill patients is typically provided by teams consisting of paramedics and a certified emergency physician. Cardiopulmonary resuscitation was performed in accordance with the European Resuscitation Council guidelines applicable at the time of data collection [19]. Accordingly in the pre-hospital management of OHCA, the placement of an SGA is part of the standard operating procedures for paramedics and is frequently performed during the initial phase of resuscitation. In line with resuscitation guidelines applicable at the time of data collection, the SGA is typically replaced by an ETT after ROSC in patients who remain comatose or require ongoing sedation and mechanical ventilation. In the participating EMS districts, endotracheal intubation was typically performed by the most experienced provider, usually the emergency physician, using continuous waveform capnography, with both direct and video laryngoscopy available [19, 20].

A structured retrospective evaluation of EMS data supplemented by data from the German Resuscitation Register was performed, covering the state capital Dresden and the districts of Gütersloh and Lippe, with a total population of approximately 1.275 million. The study period ranged from January 1, 2019, to December 31, 2021.

The present analysis constitutes a subgroup evaluation of previously published data on the performance of post-resuscitation PHEA [5, 6]. This analysis was conducted post hoc and was not prespecified in the original study protocol. The subgroup analysis was covered by the original ethics approval. The study size was determined by the number of eligible cases available within the predefined study period. No formal sample size calculation was performed. The study is reported in accordance with the STROBE statement.

### Selection of participants

All EMS missions within the specified districts and time frame were screened.

Patients aged 18 years or older with out-of-hospital, non-traumatic cardiac arrest, who were resuscitated according to the Utstein criteria (chest compressions and/or defibrillation), who were unconscious following ROSC, who had a spontaneous circulation upon hospital admission, and in whom an SGA was changed to an ETT in the pre-hospital setting were included.

Patients without OHCA, or who have not undergone resuscitation measures, for example as part of a do-not-resuscitate-order, with OHCA as a result of pre-hospital induction of anaesthesia and with missing analysis data were excluded. OHCA as a result of pre-hospital induction of anaesthesia was defined as cardiac arrest occurring in direct temporal and clinical association with the induction of anaesthesia or airway management, including drug-related effects and procedure-related events and not attributable to a primary underlying cardiac arrest.

### Measurements, definitions and exposure

Age, sex, pre-existing illness, initial ECG rhythm, bystander CPR performed, witnessed arrest, SGA applied, endotracheal intubation performed, airway complication, re-arrest, hypotension, antihypotensive therapy, at least one haemodynamic complication were recorded.

Pre-existing illness was defined as any documented history of cardiac, pulmonary, metabolic, oncological, neurological disease, or immunodeficiency.

Post-resuscitation anaesthetics were recorded, including the analgesics fentanyl and morphine, the hypnotics propofol, midazolam, ketamine, and s-ketamine, as well as the neuromuscular blocking agents rocuronium, cis-atracurium, and succinylcholine, together with their cumulative doses.

Re-arrest was defined as at least one change in heart rhythm with loss of palpable pulse following any ROSC with the need for chest compressions. Hypotension was defined as at least one systolic blood pressure of less than 100 mmHg. Antihypotensive therapy was defined as administration of theodrenaline/cafedrine and/or noradrenaline and/or adrenaline in the absence of cardiac arrest after airway exchange or, in patients receiving PHEA, after induction. An airway complication was an observed aspiration and/or difficult intubation, defined as more than one attempt for endotracheal intubation and/or difficult airway access and/or change in airway management strategy with performance of a cricothyroidotomy or reinsertion of the SGA. PHEA was defined as the administration of at least one analgesic, hypnotic, or neuromuscular blocking agent.

We compared patients with an SGA who received PHEA for endotracheal intubation with patients with an SGA who underwent endotracheal intubation without PHEA.

### Outcomes

The primary endpoint was the occurrence of at least one haemodynamic complication, defined as re-arrest and/or hypotension and/or antihypotensive therapy.

In addition, the drugs used for PHEA were listed according to the substance groups and the cumulative doses were indicated.

### Statistical analysis

For statistical analysis, propensity score matching was conducted to ensure an appropriate balance of covariates between the group with and without PHEA. This approach was chosen to maximise comparability between groups, acknowledging the trade-off of a reduced sample size due to matching. The propensity score was estimated using logistic regression including the covariates given in Table 2, followed by 1:1 matching. Covariates were selected based on clinical relevance and their established association with post-resuscitation outcomes, including age, sex, pre-existing illness, shockable initial rhythm, bystander CPR, and witnessed arrest. These variables were considered potential confounders influencing both the likelihood of receiving PHEA and the risk of post-resuscitation complications. To identify best matching partners, we used a calliper width of 0.2 standard deviations of the logit-transformed propensity score. Balance of covariates was assessed using z-differences. Absolute z-differences $\:\ge\:$ 1.41 were considered abnormal [21]. To quantify the effect of PHEA on the different endpoints, odds ratios (ORs) were estimated, along with their corresponding Wald 95%-confidence intervals. For this, a stratified logistic regression was used after successful matching [21]. All analyses were conducted using SAS 9.4 (SAS Institute Inc, Cary, NC).

### Declaration of generative AI and AI-assisted technologies in the writing process

During the preparation of this manuscript, the authors used ChatGPT and DeepL for language editing and translation assistance. After using these tools, the authors carefully reviewed and edited the content and take full responsibility for the integrity and accuracy of the manuscript.

## Results

Among 391,305 EMS missions, 2,305 OHCA were recorded (0.59%). A ROSC upon hospital admission was recorded in 706 (30.6%) patients. An SGA was initially used in 297 patients (42.1%); in 242 (81.5%) of these the SGA was changed to an ETT. This procedure was performed by an emergency physician in 211 cases (87.2%). In addition, PHEA was administered to 153 (63.2%) of these patients and at least one haemodynamic complication occurred in 134 of 242 cases (55.3%).

**Fig. 1:**
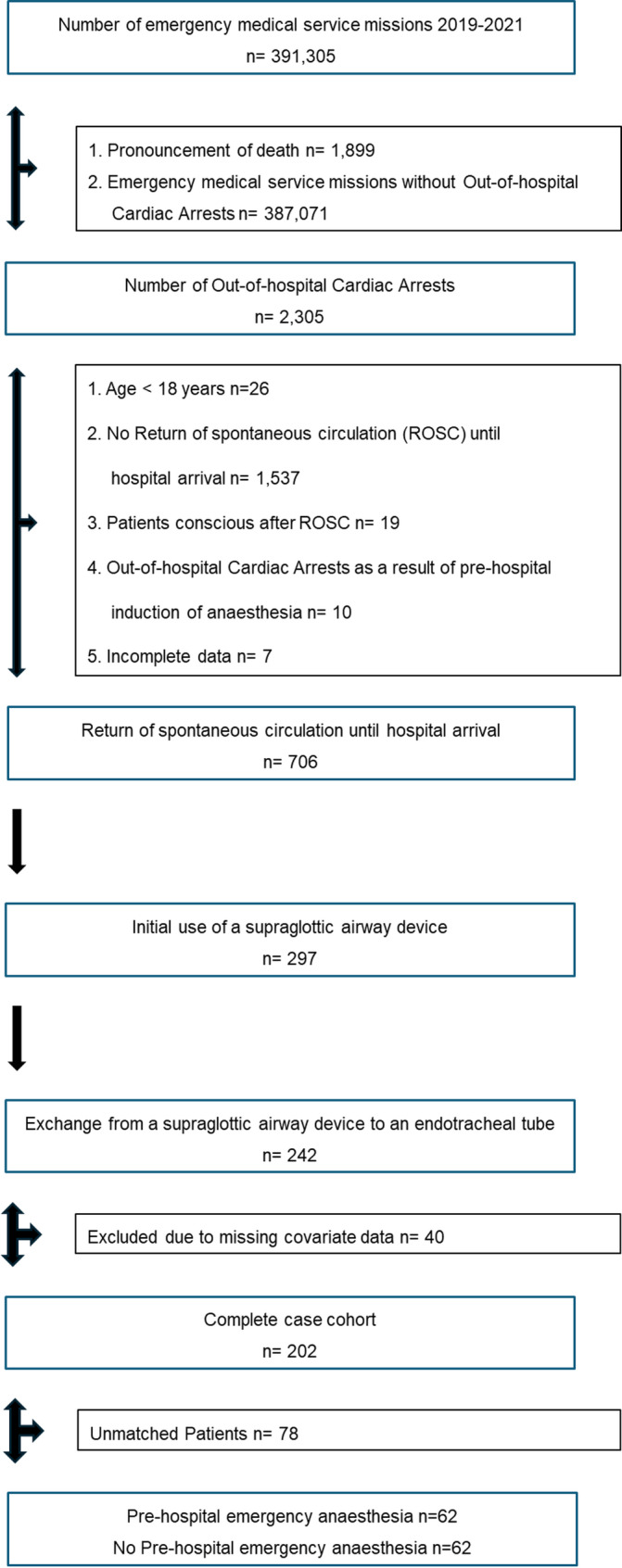
Flow diagram of patient selection and derivation of matched cohort. Numbers of eligible, excluded and analysed patients are shown

Of the 242 patients in whom an SGA was changed to an ETT, 202 had complete data for all covariates and were evaluated in the propensity score analysis (see Fig. 1). Of these, *n* = 126 (62.4%) received post-resuscitation PHEA. Missing data were primarily related to documentation of pre-existing illness (*n* = 31), while missing data for other covariates were rare (shockable rhythm *n* = 6; age *n* = 2; sex *n* = 1; antihypotensive therapy *n* = 1; succinylcholine *n* = 1). A detailed distribution of documented pre-existing illness categories, allowing for multiple categories per patient, is provided in Supplementary Table 1.

Table 1 summarises the anaesthetic agents administered for PHEA prior to propensity score matching, thereby characterising exposure patterns within the cohort. As drug selection and dosing may influence haemodynamic stability, the distribution of hypnotics, analgesics, and neuromuscular blocking agents is presented descriptively. Hypnotics were used most frequently (95.2%), followed by analgesics (56.4%) and neuromuscular blocking agents (42.9%).

**Table 1:** Drugs used for pre-hospital emergency anaesthesia before propensity score matching

Substance	*n* (%)	MCD^1^ ± SD in mg
*Fentanyl*	68 (54.0)	0.25 ± 0.15
*Morphine*	5 (4.0)	13 ± 7
*Midazolam*	95 (75.4)	11 ± 6
*Propofol*	44 (34.9)	146 ± 109
*S-Ketamine*	5 (4.0)	63 ± 50
*Ketamine*	1 (0.8)	75 ± n.a.
*Rocuronium*	30 (23.8)	64 ± 23
*Cis-Atracurium*	10 (8.0)	11 ± 3
*Succinylcholine*	16 (12.7)	104 ± 28

^1^Mean cumulative dose applied during the entire Emergency Medical Service missions

Number (proportion); Mean cumulative dose ± Standard deviation

Table 2 displays patient characteristics before propensity score matching, while Table 3 shows them after matching. Before propensity score matching, patients receiving PHEA more frequently exhibited characteristics associated with a more favourable resuscitation profile, including a higher proportion of shockable initial rhythms and witnessed arrest. Other baseline characteristics showed smaller and less consistent differences between groups. Matching reduced baseline imbalances across covariates, as reflected by lower absolute standardized Z-differences after matching in Table 3.

**Table 2:** Patient characteristics before propensity score matching

Variable	Pre-hospital emergency anaesthesia (*n* = 126)	No Pre-hospital emergency anaesthesia (*n* = 76)	z-difference
*Age*	69.3 (13.05)	67.1 (15.14)	1.01
*Sex (female)*	47 (37.3)	22 (28.9)	1.24
*Pre-existing illness* ^*2*^	114 (90.5)	69 (90.8)	-0.07
*Shockable initial ECG rhythms*	64 (50.8)	19 (25.0)	3.85
*Witnessed arrest*	102 (81.0)	48 (63.2)	2.77
*Bystander CPR*	57 (45.2)	36 (47.4)	-0.29

^2^Known cardiac, pneumological, metabolic, oncological, neurological disease

Mean (SD); Number (proportion)

**Table 3:** Patient characteristics after propensity score matching

Variable	Pre-hospital emergency anaesthesia (*n* = 62)	No Pre-hospital emergency anaesthesia (*n* = 62)	z-difference
*Age (mean)*	67.4 (12.85)	69.0 (13.97)	-0.67
*Sex (female)*	16 (25.8)	21 (33.9)	-0.99
*Pre-existing illness* ^*3*^	60 (96.8)	57 (91.9)	1.28
*Shockable initial ECG rhythm*	24 (38.7)	19 (30.6)	0.95
*Witnessed arrest*	46 (74.2)	44 (71.0)	0.40
*Bystander CPR*	36 (58.1)	29 (46.8)	1.27

^3^Known cardiac, pneumological, metabolic, oncological, neurological disease

Mean (SD); Number (proportion)

The results of the propensity score analysis are shown in Table 4. There was no evidence for differences in the odds of experiencing haemodynamic complications between patients with or without post-resuscitation PHEA, including each individual complication. Hypotension was the most common complication, followed by antihypotensive therapy and re-arrest, whereas airway complications were rare.

**Table 4:** Results of the propensity score analysis

Endpoint	Pre-hospital emergency anaesthesia (*n* = 62)	No Pre-hospital emergency anaesthesia (*n* = 62)	Odds ratio	95% confidence interval	*p*-value
At least one haemodynamic complication	40 (64.5%)	34 (54.8%)	1.50	0.72–3.11	0.277
*Re-Arrest*	9 (14.5%)	17 (27.4%)	0.39	0.14–1.08	0.069
*Hypotension*	38 (61.3%)	33 (53.2%)	1.39	0.68–2.83	0.371
*Antihypotensive therapy*	38 (61.3%)	31 (50.0%)	1.58	0.77–3.26	0.213
*Airway complication*	4 (6.5%)	4 (6.5%)	1.00	0.25–4.00	1.000

*Number (proportion)*

## Discussion

This multicentre observational study used a propensity score analysis to examine complications following post-resuscitation pre-hospital emergency anaesthesia to facilitate changing a supraglottic airway device to an endotracheal tube. There was no evidence for an association between PHEA and the occurrence of at least one haemodynamic complication, airway complications, re-arrest, hypotension, or antihypotensive therapy.

The present analysis represents a post hoc subgroup evaluation of a previously published multicentre cohort study on post-resuscitation PHEA. While the original publication focused on overall complications and outcomes after return of spontaneous circulation, the present study specifically addresses the distinct and potentially haemodynamically vulnerable situation of airway conversion from an SGA to an ETT.

### Airway strategy after ROSC

While airway management during resuscitation has been the subject of discussions in recent years, the guidelines for post-resuscitation therapy recommend changing to an ETT at an early stage following the use of an SGA, provided that the user has sufficient experience [3, 4, 17, 22].

In the post-resuscitation phase, endotracheal intubation provides protection against aspiration of gastric contents, especially regarding the high likelihood of chest compressions being performed in case of re-arrest [23]. Furthermore, it is more suitable for achieving the oxygenation and ventilation targets of post-resuscitation therapy recommended in the guidelines, particularly when increased airway pressures are required in the pre-hospital setting [3].

On the other hand, there is a risk of an increased incidence of difficult intubation in emergency care [24].

### PHEA trade-offs in the post-resuscitation setting

While studies from perioperative and intensive care medicine show that general anaesthesia and the use of neuromuscular blockers improve intubation conditions, evidence on pre-hospital emergency anaesthesia is mainly derived from observational data and remains heterogeneous [11, 25, 26]. Current guidelines emphasise that PHEA can be delivered safely when performed by experienced providers within structured systems, while also recognising the increased risk of haemodynamic instability following induction, especially in post-ROSC patients [26].

The present study shows that following ROSC, PHEA is often delivered to facilitate exchange of an SGA for an ETT. It therefore seems sensible to consider the optimal execution of PHEA for this procedure. While the guidelines on emergency anaesthesia and airway management in critically ill patients do not contain any specific recommendations on the optimal use of different combinations of anaesthetic agents, the ERC resuscitation guidelines recommend the use of a low-dose sedative and analgesic as well as a fast-acting neuromuscular blocking drug [3, 4, 17, 22].

Although post-resuscitation PHEA has been shown to have beneficial effects with improved oxygenation and ventilation after airway management, potential complications of anaesthesia used such as hypotension and re-arrest may disfavour its use in the context of airway exchange [5, 6]. Concerns about complications in this particularly critical phase after ROSC may explain why 1/3 of these procedures were performed without delivering PHEA [27]. However, this approach should be balanced against the potential for conscious awareness during and after resuscitation, which has been reported in a subset of survivors and may be associated with distressing experiences [8]. In addition, minimising secondary brain injury is a central goal of post-resuscitation care. Adequate sedation and analgesia during invasive airway interventions may therefore be clinically relevant in this vulnerable phase, as they may reduce stress responses and cerebral metabolic demand, and contribute to seizure control [28, 29]. However, awareness, psychological outcomes, and potential neuroprotective effects of PHEA were not assessed in the present study, and these aspects should be interpreted with caution.

### Airway complications

Airway complications, such as difficult intubation or aspiration, occur significantly more often with rapid sequence induction (RSI) outside the operating room, with a probability of 8–12%, than under controlled conditions [24]. In the present study, the probability of airway complications was 6.5% and thus comparable to previously reported rates, despite the less controlled environmental and organisational conditions of the pre-hospital setting. This may partly be explained by a structured approach to airway management, with standardised RSI protocols and provider experience likely contributing to safe and efficient intubation [30, 31].

### Circulatory complications

While in pre-hospital RSI without prior OHCA, around 3% of patients suffer a cardiac arrest, the present study showed a pre-hospital re-arrest probability of 21.0% following the procedure of changing an SGA to an ETT, which is consistent with current literature (10.2–40%) [32, 33].

The probability of re-arrest may be explained by the increased susceptibility to arrhythmic events in this patient population. In addition to myocardial dysfunction in the context of post-cardiac-arrest syndrome, the use of potentially arrhythmogenic substances such as adrenaline could also play a role [34, 35].

In the present study, the prevalence of hypotension was 57.3% and thus in the range of the known values for hypotension in OHCA patients (50.7–54.2%) [36]. In line with this, antihypotensive therapy was administered in 55.6% of cases, reflecting both the haemodynamic vulnerability of this population and current guideline recommendations to avoid hypotension after ROSC [3]. The incomplete overlap with the observed hypotension rate suggests that vasoactive support may have been used both therapeutically and pre-emptively, particularly when agents with pronounced haemodynamic effects such as propofol were administered.

In addition to myocardial dysfunction associated with post-cardiac-arrest syndrome and the underlying causes of shock in OHCA, anaesthetic drugs may further aggravate haemodynamic instability, particularly when administered in doses that exceed the immediate clinical requirement [37]. Although not explicitly investigated, the decision to perform PHEA, as well as agent selection and dosing, should be balanced against the patient’s haemodynamic condition in the post-resuscitation phase [11, 26].

### Anaesthetic regimen in the post-resuscitation setting

The anaesthetic regimen observed in this study is broadly consistent with current emergency anaesthesia guidelines and reflects usage patterns reported in other studies on patients undergoing rapid sequence induction. Notable differences, however, were observed in the frequency of midazolam use (75.4% in the present study vs. 36.4% reported by Russotto et al.), which may be explained by a reduced reliance on propofol due to concerns about its potential to induce hypotension [38]. The lower rate of neuromuscular blocking agent administration (44.5% in the present study vs. 75.5% reported by Russotto et al.) may reflect variation in real-world clinical practice in the pre-hospital post-resuscitation setting, where airway management is adapted to the clinical context and may not fully correspond to standardised RSI protocols [13, 16, 38].

Based on favourable pharmacokinetics and a minimal incidence of adverse drug reactions, the use of midazolam or ketamine as hypnotic agents, fentanyl or sufentanil as analgesics, and succinylcholine or rocuronium as neuromuscular blocking agents implemented within a post-resuscitation management bundle that includes pre-emptive catecholamine therapy, may represent a pragmatic approach.

### Limitations

In addition to the typical limitations of retrospective studies, this observational study is subject to potential selection bias. It is possible that post-resuscitation PHEA may have been administered preferentially to patients who were perceived as less critically ill by the treating emergency team. Furthermore, baseline haemodynamic status and the timing of vasopressor therapy may have influenced both treatment decisions and outcomes but were not consistently available in the dataset. In addition, haemodynamic assessment was based on non-invasive blood pressure measurements, which may be less accurate in critically ill patients and could have influenced the detection and classification of hypotension [39]. As a result, residual confounding and indication bias cannot be excluded.

A further limitation relates to missing data, particularly for pre-existing illness. This likely reflects incomplete documentation in routine EMS practice, where data recording occurs under time-critical conditions and may not capture all relevant clinical information. As a result, exclusion of these cases may have introduced selection bias, and the results should therefore be interpreted with caution, as missing data may have led to residual confounding. In addition, missing data in several baseline variables limited the extent to which a more comprehensive description of patient characteristics could be provided.

Furthermore, only patients admitted to hospital with ROSC were included. Patients who may have experienced fatal complications before hospital admission were not captured, which may have introduced survivor bias. This could potentially lead to an underestimation of complications associated with post-resuscitation PHEA. These findings may therefore primarily apply to a clinically relevant subgroup of patients with sustained ROSC and relative haemodynamic stability. The impact of pre-hospital mortality on the association between PHEA and outcome is currently being investigated in a separate study using an independent dataset.

Propensity score matching reduced the sample size to 62 matched pairs. While this approach improves covariate balance and reduces confounding, it may have limited statistical power to detect small to moderate effect sizes and may affect the generalisability of the findings. Given the resulting wide confidence intervals, clinically relevant differences between groups cannot be excluded. Accordingly, the findings should be interpreted with caution and do not allow firm conclusions regarding the presence or absence of an effect of post-resuscitation PHEA.

In addition, patient-relevant long-term outcomes such as neurological status or mortality could not be assessed, precluding conclusions regarding long-term benefit. Pre-hospital post-resuscitation management was not standardised. Therefore, the influence of specific interventions, such as the use of video laryngoscopy or pre-emptive catecholamine therapy prior to induction, cannot be determined.

## Conclusions

In the context of pre-hospital emergency care of post-resuscitation patients, PHEA is often performed to facilitate the replacement of a supraglottic airway with an endotracheal tube. The present study showed no evidence for an association between the administration of PHEA and the occurrence of haemodynamic or airway complications. Future studies should investigate different procedures for PHEA in order to develop recommendations for future guidelines.

## Supplementary Information

Below is the link to the electronic supplementary material.

Supplementary Material 1

## Data Availability

The datasets generated and analysed during the current study are not publicly available due to data protection regulations but are available from the corresponding author on reasonable request.

## Abbreviations

OHCA: Out-of-hospital cardiac arrest
PHEA: Pre-hospital emergency anaesthesia
SGA: Supraglottic airway device
ETT: Endotracheal tube
ROSC: Return of spontaneous circulation
EMS: Emergency Medical Services
RSI: Rapid sequence induction
