# Supplementary material for: Post-resuscitation pre-hospital emergency anaesthesia for exchange of a supraglottic airway device with an endotracheal tube - a multicentre observational study
Source: BMC Emerg Med. 2026 Jul 13;26:194. doi: 10.1186/s12873-026-01668-8 (PMC13366920; doi:10.1186/s12873-026-01668-8)
Supplement: Supplementary file 1 — Supplementary Material 1 [file 12873_2026_1668_MOESM1_ESM.docx]

Supplementary Table 1: Distribution of pre-existing illness categories. Number (proportion)^[[1]](#footnote-1)^.

| **Variable** | **Included**  **(n=222)** | **Pre-hospital emergency anaesthesia**  **(n=145)** | **No pre-hospital emergency anaesthesia**  **(n=77)** |
| --- | --- | --- | --- |
| Cardiac | 93 (41.9 %) | 65 (44.8 %) | 28 (36.4 %) |
| Pulmonary | 34 (15.3 %) | 24 (16.6 %) | 10 (13.0 %) |
| Metabolic | 38 (17.1 %) | 25 (17.2 %) | 13 (16.9 %) |
| Oncological | 12 (5.4 %) | 7 (4.8 %) | 5 (6.5 %) |
| Neurological | 16 (7.2 %) | 9 (6.2 %) | 7 (9.1 %) |
| Immunodeficiency | 1 (0.5 %) | 0 (0.0 %) | 1 (1.3 %) |
| Other / unspecified pre-existing illness^[[2]](#footnote-2)^ | 28 (12.6 %) | 15 (10.3 %) | 13 (16.9 %) |

1. Patients could have more than one documented pre-existing illness category. Percentages are based on the total number of documented pre-existing illness categories in the respective group. A total of 222 documented pre-existing illness categories were identified among 183 included patients with pre-existing illness, including 145 categories among 114 patients receiving pre-hospital emergency anaesthesia and 77 categories among 69 patients not receiving pre-hospital emergency anaesthesia. [↑](#footnote-ref-1)
2. Documented pre-existing condition without further specification in the EMS record. [↑](#footnote-ref-2)
